# Validation of behavioral measures of social cognition in individuals diagnosed with schizophrenia

**DOI:** 10.3389/fpsyg.2024.1443145

**Published:** 2024-09-09

**Authors:** Noa Rahamim, Reut Gilad, Omer Linkovski, Hagai Bergman, Keren Avirame, Yasmin Abo Foul, Renana Eitan

**Affiliations:** ^1^The Edmond and Lily Safra Center for Brain Science, Hebrew University, Jerusalem, Israel; ^2^Psychiatric Division, Tel Aviv Sourasky Medical Center Ichilov, Tel Aviv, Israel; ^3^The Jerusalem Mental Health Center, Jerusalem, Israel; ^4^Department of Psychology, Bar-Ilan University, Ramat Gan, Israel; ^5^The Gonda Multidisciplinary Brain Research Center, Bar-Ilan University, Ramat Gan, Israel; ^6^Department of Medical Neurobiology, Institute of Medical Research Israel-Canada (IMRIC), Hadassah-Hebrew University Medical School, Jerusalem, Israel

**Keywords:** schizophrenia, social cognition, theory of mind (ToM), emotion recognition, comic strip, Montreal Affective Voices (MAV), Go-NoGo

## Abstract

Schizophrenia, a complex neuropsychiatric disorder, manifests severe impairments in social cognition, notably in Theory of Mind (ToM), empathy, and emotion recognition, which significantly influence social competence and overall functioning. These aspects are crucial for prognosis in individuals diagnosed with schizophrenia (SZ). This study validates a comics strip paradigm for ToM and empathy assessment, the Montreal Affective Voices (MAV) for measuring emotion recognition, and a Go-NoGo task for inhibition control estimation in individuals diagnosed with SZ, comparing their performance with healthy controls. SZ participants exhibited diminished abilities in the comics strip task, especially in ToM and empathy conditions, alongside challenges in identifying emotions from vocal cues in MAV. They responded slower and tended to be less accurate in the Go-NoGo task. The validated behavioral battery addresses the limitations of previous measures and emerges as a promising tool for future investigations into the neural systems underlying social cognition in schizophrenia. Such insights can lead to the development of long-needed treatment for negative symptoms and social dysfunctions in schizophrenia.

## Introduction

Schizophrenia is a chronic complex neuropsychiatric disorder that affects ~1% of the world’s population (Kahn et al., 2015). The Diagnostic and Statistical Manual of Mental Disorders (DSM-5) diagnosis of schizophrenia involves a constellation of five core symptoms: delusions, hallucinations (together termed positive symptoms), disorganized speech, disorganized behavior, and negative symptoms (affective flattening, avolition, alogia, anhedonia, and asociality), with impaired occupational or social functioning (American Psychiatric Association, 2013). As in several neuropsychiatric conditions, social cognition is severely impaired in schizophrenia (Bellack et al., 1990). The positive symptoms of schizophrenia typically emerge in the early to mid-20s in males and the late 20s in females (Van Der Werf et al., 2014), and can be controlled in most patients by antipsychotic medications (Haddad and Correll, 2018). The onset may be abrupt, but most patients manifest a prodromal phase with gradual development of positive and negative symptoms (Klosterkötter, 2008). Once diagnosed, remission is rare, and individuals remain chronically ill (Robinson et al., 2004). The treatment-resistant deficits in social cognition (Swartz et al., 2007) are strongly associated with functional outcomes of the disease (Couture et al., 2006; Fett et al., 2011). It was recently suggested that improvement of the impaired social functioning observed in schizophrenia can be achieved by targeting neural systems that underlay social cognition (Dodell-Feder et al., 2015). The first step is to develop the appropriate tools that will allow the identification of these underlying neural systems while measuring impaired social functions.

One of the main components of social cognition is emotion recognition, the ability to encode the emotional state of others from sensory stimuli containing emotional information (Ferretti and Papaleo, 2019). Emotion recognition tasks are the most extensively used paradigms to assess human social cognition, mostly focusing on facial expression (Ferreira et al., 2021) and emotional prosody (Buchanan et al., 2000; Pell, 2006). Several meta-analysis reviews of studies on emotional prosody in schizophrenia indicated that individuals with schizophrenia are impaired in the perception and expression of emotional prosody (Hoekert et al., 2007; Lin et al., 2018). Most of the studies described in these reviews use spoken words or sentences with a variety of emotional tones. However, claims have been raised as to the complexity of such tasks, which potentially involve the linguistic function of prosody and the semantic content provided by language (Scherer et al., 1984). To overcome this pitfall Belin et al. (2008) created the Montreal Affective Voices (MAV), a validated set of auditory nonverbal affective stimuli (Belin et al., 2008). Using five out of the eight emotion categories from the MAV Kraus et al. (2019) showed auditory processing deficits associated with auditory emotion recognition in schizophrenia.

Another key aspect of social cognition is Theory of Mind (ToM), the ability to model the internal mental state of other individuals, including thoughts, beliefs, intentions, desires, and emotions, and to use it to explain and predict their behavior (Carlson et al., 2013; Frith and Frith, 2005). ToM is known to be severely impaired among individuals with schizophrenia (Savla et al., 2013), and some even claim that the behavioral symptoms of schizophrenia might be best understood in the context of this disturbance (Frith, 2014). While most consider ToM to be a single construct, some distinguish between affective and cognitive ToM (van Neerven et al., 2021). This distinction leads to the connection between ToM and empathy. A basic definition of empathy is the ability to understand and share the feelings of another. However, the term has an extensive history and many alternative definitions (Cuff et al., 2016). The complexity is further demonstrated by the division of the empathy concept into “cognitive empathy” which emphasizes the cognitive and intellectual processes (e.g., accurate perception), and “affective empathy” which refers to the capacity to experience affective reactions to the observed experience of others (Shamay-Tsoory, 2013). According to this view, cognitive empathy involves ToM (Shamay-Tsoory et al., 2004). The segregation of both ToM and empathy into “cognitive” and “affective” domains reflects the lively debate around these two terms, and their respective parallel theoretical frameworks—“theory theory” (TT) and “simulation theory” (ST) (Alcalá-López et al., 2019). While some use these terms interchangeably (Baron-Cohen et al., 2001) others claim they can be discriminated at the level of neural networks and cerebral structures (Grèzes et al., 2004; Lamm et al., 2011; Shamay-Tsoory et al., 2009).

Social cognition deficits are widely investigated in schizophrenia. While a clear association is found between schizophrenia and TT deficits (manifested as impairment in the abstract ability to infer other’s intentions and beliefs) the effects on ST are less clear (Alcalá-López et al., 2019). Several studies reported preserved ST capacity and even enhanced simulated affective states in individuals with schizophrenia (Achim et al., 2011; Michaels et al., 2014; Horan et al., 2016). Conversely, Shamay-Tsoory et al. (2007) found that schizophrenia is associated with a significant impairment in both cognitive and affective empathy. Furthermore, a meta-analysis by Bonfils et al. (2016) showed significant medium affective empathy deficits in schizophrenia. With evidence to support both sides of the debate, Apperly (2008) claimed that the data obtained from neuroimaging studies did not provide true discrimination between ST and TT. In the author’s view, the problem lies in the conceptual debate, and a possible solution would be to identify which neural systems are involved in social cognition processes. Even with the contradicting evidence in the debate over the discriminability of ToM and empathy these social cognition functions are affected in individuals with schizophrenia. Moreover, it was shown that these social cognition deficits, specifically ToM, are domain-specific and are not a consequence of general cognitive impairment of attention, executive functioning, memory, and general intelligence (Langdon et al., 2001).

The effect of social cognitive deficits and negative symptoms on the daily life functioning of individuals with schizophrenia is intertwined (Madeira et al., 2016). Together, these constructs predict social competence and functioning in people with schizophrenia (Kalin et al., 2015), and are more related to prognosis than positive symptoms (Tamminga et al., 1998; Kotov et al., 2017). Yet, to date, no effective treatment for the negative symptoms of schizophrenia is available (Galderisi et al., 2021), and while positive symptoms are mostly well treated and diminish over the life course, the negative symptoms tend to be persistent and disabling throughout the lifespan of schizophrenia (Millan et al., 2014). Finding a new therapeutic approach to improve social functioning and deal with the negative symptoms of schizophrenia is long-needed and overdue. Deep brain stimulation (DBS) is a new therapeutic approach in treatment-resistant neuropsychiatric patients (Graat et al., 2017). In many of the disorders, the basal ganglia constitute the target for therapeutic stimulation (Malone, 2010; Alonso et al., 2015; Baldermann et al., 2016). Previous studies exploring the neural anatomy, biology, and physiology of social cognition stressed the critical role of the basal ganglia in social cognition functions (Bodden et al., 2010; Christidi et al., 2018) and social behaviors (Adolphs, 2001). Thus, there are reasons to hypothesize that DBS of the basal ganglia could serve as a novel treatment for the negative symptoms and social cognition impairments of schizophrenia.

Our goal was to create a battery of behavioral tasks that can measure social cognition functions in subjects diagnosed with schizophrenia and distinguish these functions from more general cognitive functions. We aimed to validate two modifications of tasks that measure social cognition in a population of individuals with schizophrenia (SZ) in Israel and compare them to healthy control (HC). We chose a validated set of comic stories to measure the aspect of ToM and Empathy (Völlm et al., 2006). We included several newly created comics in a matching style to expand the set, increasing the number of trials without comic repetition. Emotion recognition was estimated using the Montreal Affective Voices (MAV) (Belin et al., 2008), using all eight emotions. Additionally, we used a modified version of the Go-NoGo task to measure inhibitory control (Trommer et al., 1988; Criaud and Boulinguez, 2013), a cognitive executive function (Diamond, 2013). The behavioral battery was designed to allow a proper electrophysiological study in the future, hoping that this battery will lead to the identification of neural systems and activity patterns underlying social cognition functions in general (e.g., discriminability between ToM and empathy), and specifically in schizophrenia. A better understanding of the pathophysiological mechanisms that underlie this disease can lead to the development of new treatments for the negative symptoms and social cognition deficits.

## Materials and methods

### Participants

Twenty-five individuals diagnosed with schizophrenia (SZ) according to DSM-5 criteria (American Psychiatric Association, 2013), and treated in the Jerusalem mental health center, participated in this study. They were recruited by a psychiatry resident (RGL) while being hospitalized or when visiting the outpatient clinic. The resident evaluated the SZ subjects’ ability to cooperate and complete the behavioral tasks properly and recruited them based on these criteria. Demographic information was collected including age, gender, and years of education (Table 1).

**Table 1:** Sample characteristics and screening tests (MMSE and BDI) by groups.

	SZ (*n* = 25)	HC (*n* = 29)
Age range (years)	22–41	20–50
Mean age (±SD)	28.28 (±5.05)	25.9 (±6.73)
Gender: female (%)	4 (16%)	15 (52%)
Education: average years (±SD)	11 (±1.71)	13.75 (±1.97)
MMSE (±SD)	25.96 (±6.03)	
BDI average (±SD)	12.24 (±7.96)	

Data from 29 healthy controls (HC) were collected. Participants were students recruited at the Hebrew University in Jerusalem and the Bar-Ilan University in Ramat-Gan, Israel. The study was approved by the Institutional Review Board of Ethical Conduct of the Hebrew University of Jerusalem and the Jerusalem Mental Health Center (IRB number 7-19).

### Procedure

All subjects volunteered to participate and signed a participation consent form. Subsequently, each subject performed several behavioral tasks, in a randomized order. Due to time constraints, not all participants completed all the tasks. The number of participants in each task is provided in Supplementary Table S2. Each task started with an explanatory computer video, demonstrating the task and its instructions. When needed, subjects completed several training trials to ensure they understood the instructions and could perform the task properly. Participants were free to take breaks between tasks as they desired. After completing the tasks, the SZ group was assessed by completing a computerized version of the Beck Depression Inventory (BDI) (Beck et al., 1961) and a Mini-Mental State Examination (MMSE) (Folstein et al., 1975) to exclude severe depression and general cognitive impairments.

### Clinical assessments

*Mini-Mental State Examination (MMSE)* (Folstein et al., 1975), a validated 30-point cognitive screening test used extensively to measure cognitive impairments (Brayne, 1998). A score of 24 or more is considered normal cognition. Below this, scores can indicate mild (19–23 points), moderate (10–18 points) or severe (≤9 points) cognitive impairment. The MMSE demonstrates reliability and has been reported to be internally consistent (Bernard and Goldman, 2010).

*Beck Depression Inventory (BDI)* (Beck et al., 1961), is a 21-item, self-report rating inventory that measures characteristic attitudes and symptoms of depression. A total score of 0–13 is considered a minimal range, 14–19 is mild, 20–28 is moderate, and 29–63 indicates severe depression. BDI has a mean internal consistency of 0.86 and a high internal consistency for both psychiatric and non-psychiatric populations, with alpha coefficients of 0.86 and 0.81, respectively (Beck et al., 1988). We created and used a computerized version of the BDI.

### Behavioral tasks

#### Comics strip

**Figure 1:**
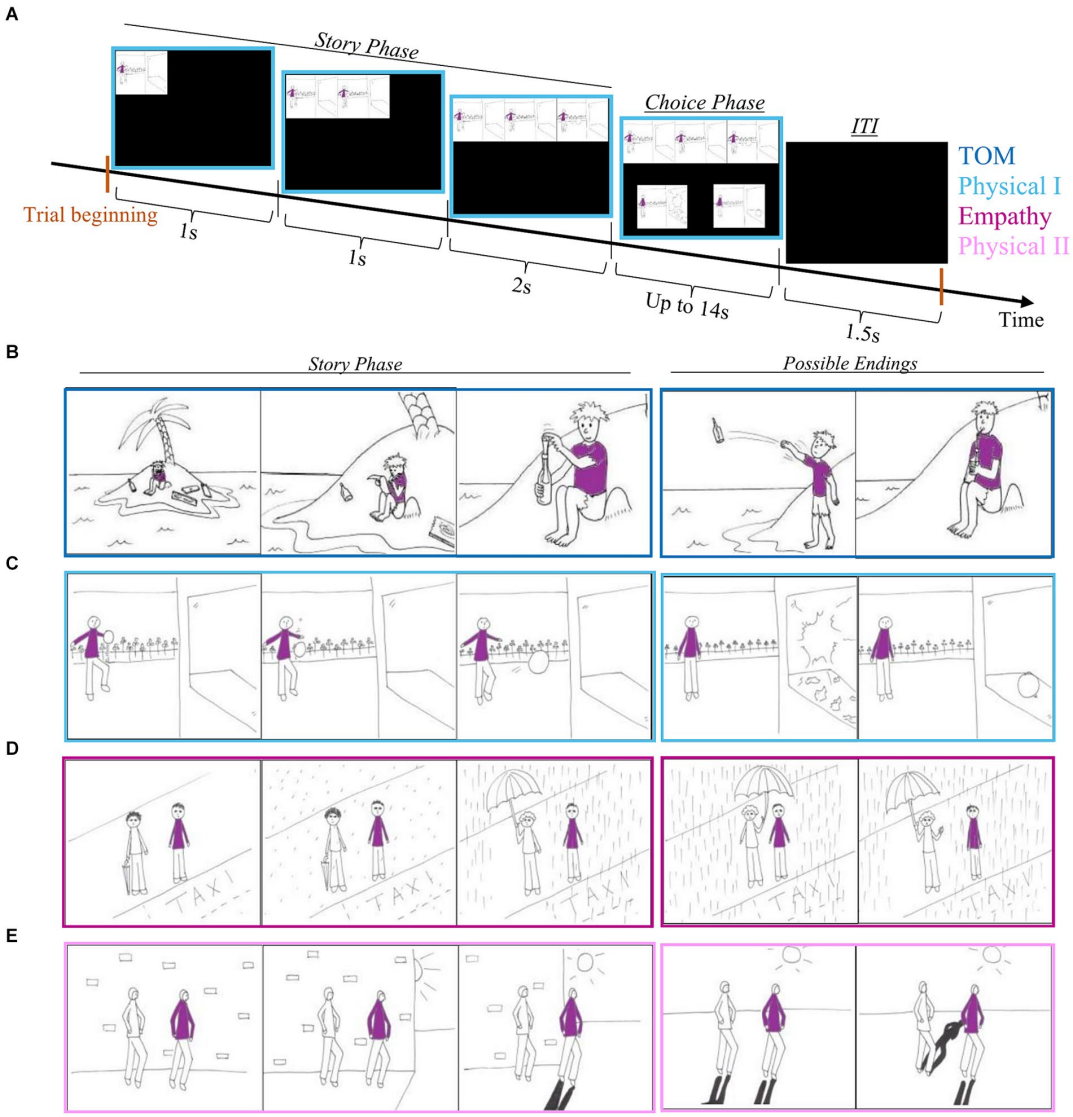
Comics strip scheme. **(A)** One trial scheme. **(B)** Example of a comic from the ToM condition. **(C)** Example of a comic from the Physica1 condition. **(D)** An example of a comic from the Empathy condition. **(E)** An example of a comic from the Physical2 condition.

Social cognition in schizophrenia can be assessed by tasks that utilize pictures depicting social situations (Brüne et al., 2008; Walter et al., 2009; Lee et al., 2010). We created and used a modification (description below) of the task by Völlm et al. (2006). The original task had 4 experimental conditions: Theory of Mind (ToM) in which the subject is required to understand the desires and intentions of the animated character; Empathy in which the subject needs to understand how one character shows empathy to another; and two physical conditions in which understanding physical causality is required. One of these conditions involved only one character (Physical1) and serves as a control for the ToM condition. The other demonstrates two characters (Physical2) and serves as a control for the Empathy condition. We used the original comics together with several new stories we created according to the original style. These new stories were added to allow enough repetitions in a future electrophysiological study. We also changed the duration of the comics presentation to ensure participants had enough time to understand the depicted story and choose the ending they deemed to be correct. The task included 52 stories (trials), 13 per condition, which were presented in three sequential comic pictures. Each trial started with the story phase in which the first picture was presented for 1 s. The first picture was followed by the addition of a second picture for another 1 s, and finally, the last picture was added. The whole story (three pictures) was presented for 2 s and was followed by the choice phase in which two possible endings to the story were presented below it. Participants were instructed to choose the correct ending to the story by pressing the button that corresponded to the presentation side on the screen. The window for response was 14 s. If the subject responded within this time frame the next trial started after an inter-trial interval (ITI) of 1.5 s. If not, the next trial started after 14 s and an ITI of 1.5 s (Figure 1).

#### Emotional voices

**Figure 2:**
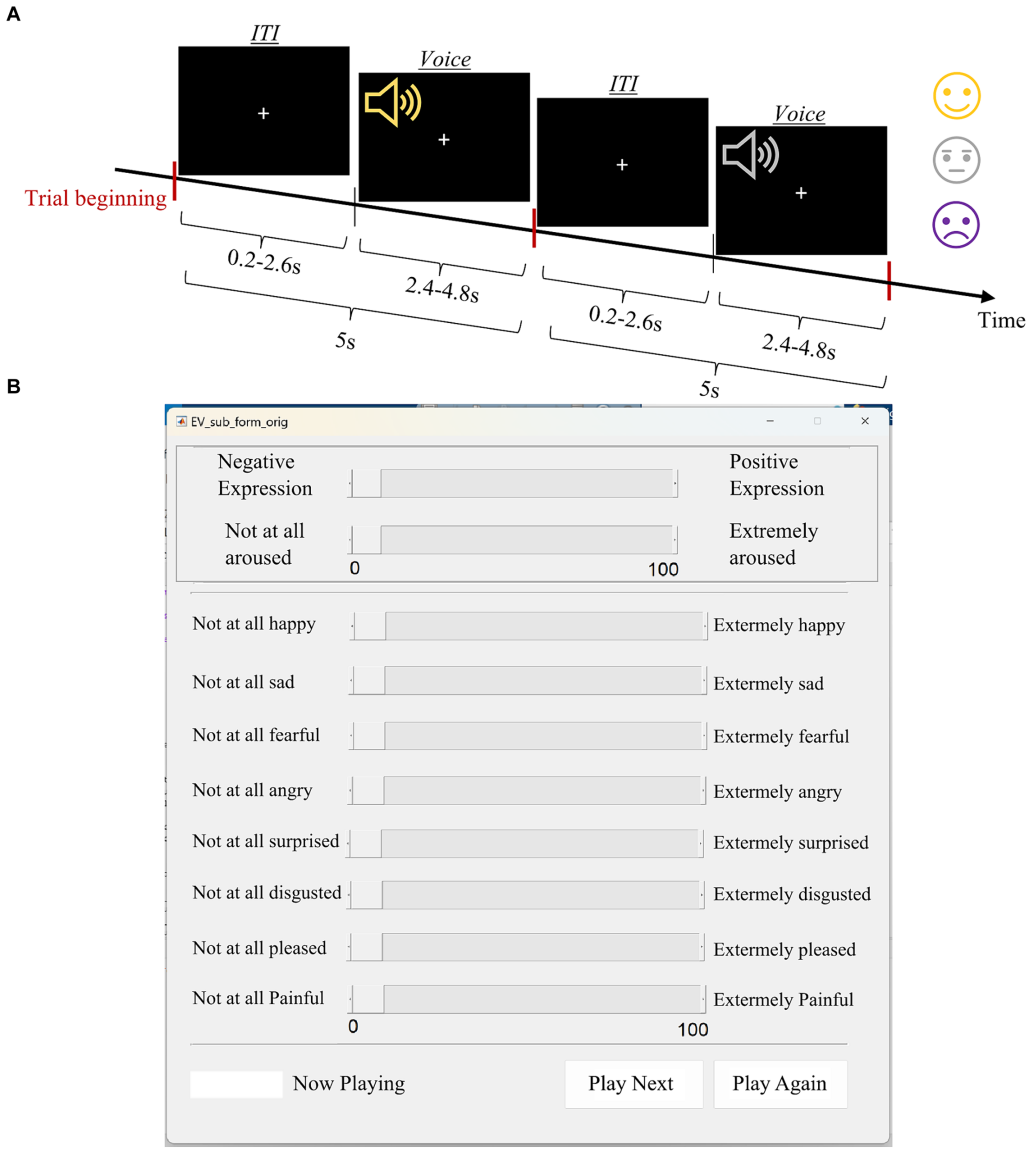
Emotional voice scheme. **(A)** One trial scheme. **(B)** Rating form.

The Montreal Affective Voices (MAV) is a validated tool for research on auditory affective processing (Belin et al., 2008), which was previously validated in Israel (Eitan et al., 2013). The MAV consists of 90 nonverbal affect bursts (using the vowel /a/ as in “apple”) corresponding to eight different emotions (anger, disgust, fear, pain, sadness, surprise, happiness, and pleasure) as well as neutral vocalizations, recorded by 10 actors (5 male and 5 female). Due to the long duration of the original task and our time limitation, we included only 49 out of the 90 voices (5-6 voices from each category) in our version of the task. The voices were presented in a pseudo-randomized order such that each trial, consisting of the voice and an inter-trial interval (ITI), lasted for 5 s (Figure 2A). Following the presentation of each voice, a computerized form was presented to the subject. The subject was instructed to state, using a scale from 0 to 100, the intensity of the emotion and whether it was more negative (0) or positive (100) (valence rating). In addition, the form contained a separate scale for each emotion, and subjects were instructed to only refer to the emotions they thought were relevant to the last voice they heard (Figure 2B). Subjects could listen to the voice as many times as they wished, by pressing the “play again” button. The number of “play-again” presses and the reaction time are recorded.

#### Go-NoGo

**Figure 3:**
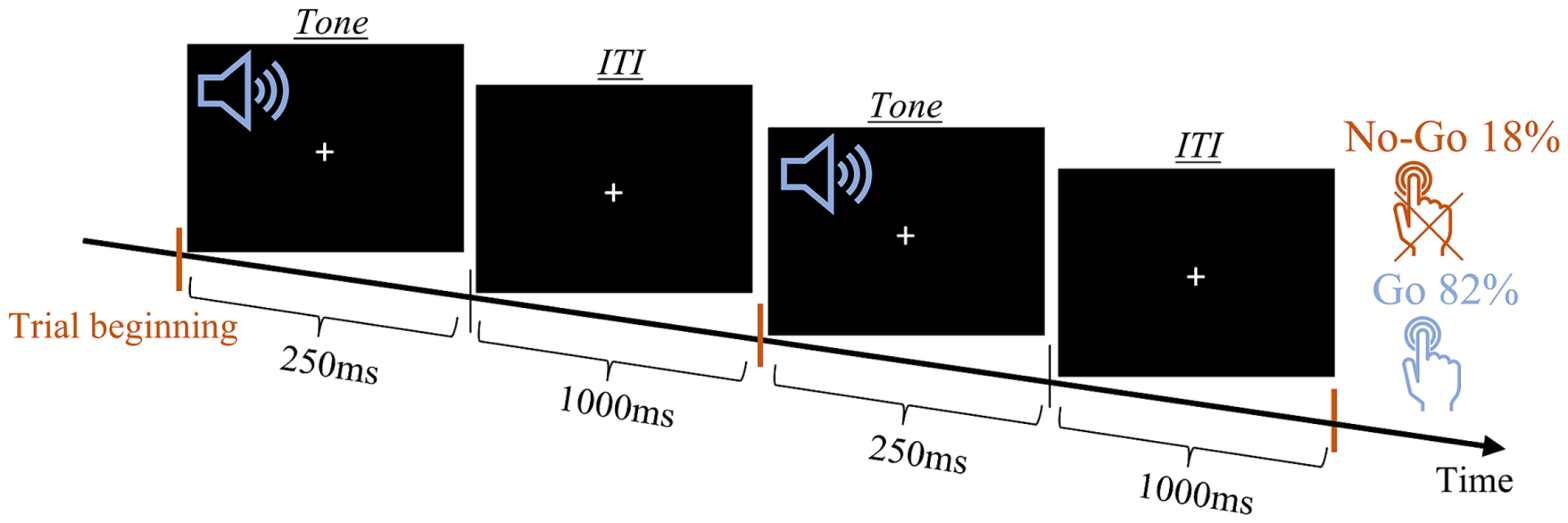
Auditory Go-NoGo task scheme. Schematic presentation of two trails. Orange lines mark the trail onset.

A variety of Go-NoGo tasks are widely used to measure the capacity for sustained attention and response control. One such version was previously validated by our group, in a study that involved Parkinson’s disease patients and included 3 variations of the task: All-Go, None-Go, and Go-NoGo (Marmor et al., 2020). Marmor et al. (2020) compared the performance of the participants in the three tasks and showed that responses were not related to the auditory properties (tone pitch) but to the meaning that was assigned to them, denoting to press the button (“Go”) or withholding the response (“NoGo”). Here, we only run the Go-NoGo task, with one of the tones played in significantly more trials (frequent tone) than the other (deviant tone). In this task, participants are instructed to respond to the frequent tone and to refrain from responding to the deviant tone. When the ratio of frequent to deviant is relatively high, and the ITI is fixed this auditory oddball task results in a rhythmic and repetitive pressing that is difficult to withhold during the deviant tones. In the current study, the frequent tone was delivered at low pitch (2,200 Hz) in ~82% percent of the trials and the deviant tone at high pitch (4,400 Hz) in the remaining ~18% of trials. In each of the 120 trials, the tone was played for 250 ms and was followed by a constant ITI of 1,000 ms (Figure 3). Participants’ response was registered using two hand buttons, one in each hand. We instructed participants to respond to the low pitch tone by pressing both buttons as fast as possible and avoid pressing the buttons after the high pitch tones.

### Analysis strategy

We analyzed the independent measures of all tasks using a mixed analysis of variance (ANOVA) with age, gender, and years of education as covariates. When required, *p*-values were adjusted using Greenhouse–Geisser correction, and Bonferroni corrections were applied for follow-up post-hoc comparisons (*t*-tests).

#### Comic strip

Two independent measures were evaluated: (1) accuracy and (2) response time (RT) of correct responses. For each subject, we calculated the accuracy in each condition by counting the number of correct answers in each condition (i.e., choosing the correct ending to the story out of two possible endings) and dividing by 13 (the number of stories in each condition). We analyzed both accuracy and RT data using a 2 (group: SZ and HC) × 4 (condition: ToM, Physical1, Empathy, and Physical2) mixed ANOVA with age, gender, and years of education as covariates.

#### Emotional voices

Three independent measures were evaluated: (1) accuracy, (2) intensity ratings, and (3) valence ratings. We defined response as correct when the subject’s ranking of the displayed emotion was the highest out of all ranked emotions. The “neutral” emotion served as a control condition and was not present in the computerized ranking form (Figure 2B). Thus, there was no correct way to rank it, and accuracy in terms of emotion identification was essentially not collected. Therefore, we excluded the rated accuracy analysis of the “neutral” condition, while including it in the intensity and valence analysis. We analyzed accuracy with a 2 (group: SZ and HC) × 8 (emotion: anger, disgust, fear, pain, sadness, happiness, pleasure, surprise) mixed ANOVA with age, gender, and years of education as covariates, and the other 2 measures using a 2 (group: SZ and HC) × 9 (emotion: Anger, Disgust, Fear, Pain, Sadness, Happiness, Pleasure, Neutral and Surprise) mixed ANOVA with age, gender, and years of education as covariates.

#### Go-NoGo

Two independent variables were measured: (1) accuracy and (2) response time (RT). As the response was registered via two buttons, one at each hand, RT was defined as the minimum of the two RTs. The effects of these two variables were tested using a 2 (group: SZ and HC) × 2 (condition: Go, NoGo) mixed ANOVA with age, gender, and years of education as covariates.

## Results

### Demographic and clinical characteristics

There were significant differences between groups in gender [female ratio; *t*(52) = 2.9, *p* = 0.005] and years of education [*t*(51) = 5.44, *p* < 0.001] (Table 1; Supplementary Table S1). BDI scores revealed no severe depression and MMSE showed preserved general cognitive abilities of participants in the SZ group. In addition, we performed a correlation analysis between the clinical measures and behavioral accuracy in all three tasks. No significant Bonferroni-corrected correlations were detected (Supplementary Tables S3, S4).

### Comics strip

#### Accuracy

**Figure 4:**
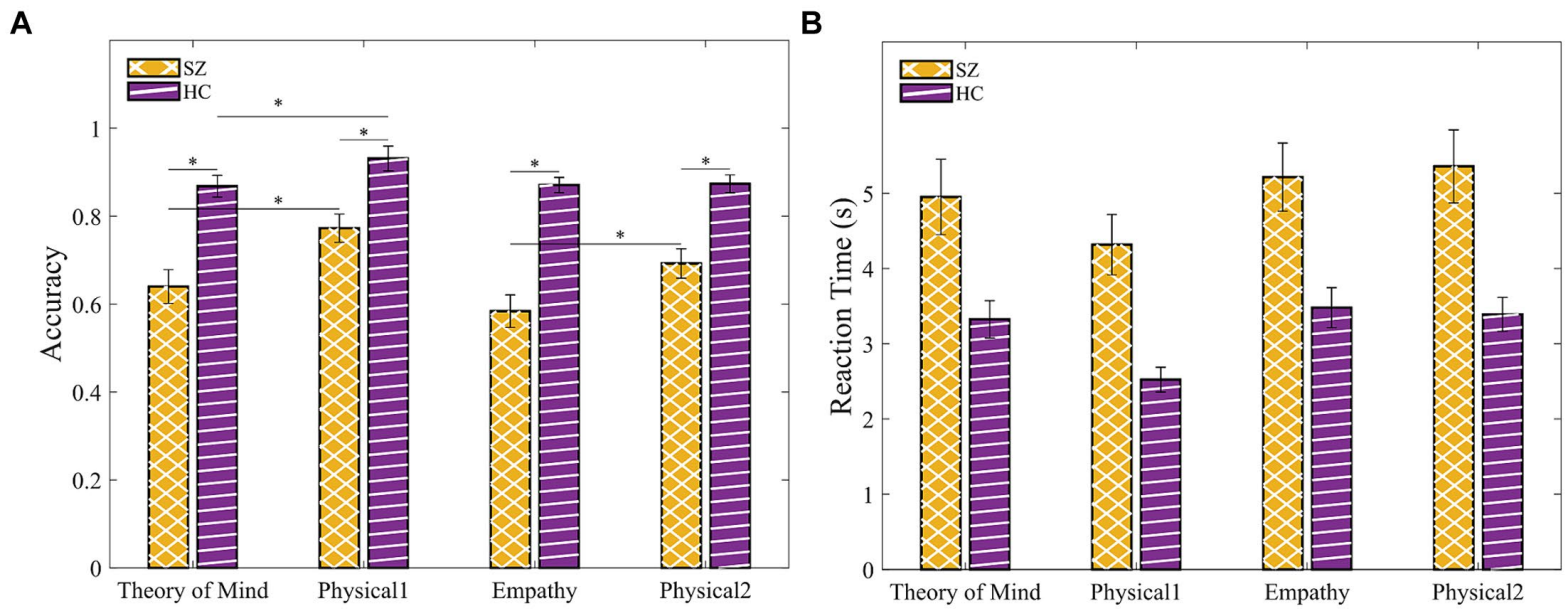
SZ shows decreased abilities in choosing the correct ending to the story particularly in the social cognition conditions. **(A)** Accuracy. **(B)** Response time. Error bars indicate the standard error of the mean (SEM). Asterisks indicate the level of statistical significance: *p < 0.001.

A 2 (group: SZ, HC) × 4 (condition: ToM, Physical1, Empathy, Physical2) mixed ANOVA with age, gender, and years of education as covariates (Supplementary Table S5) was conducted. We found a significant main effect of group [*F*(1, 43) = 12.31, *p* = 0.001, η^2^*_p_* = 0.22] and a significant group × condition interaction [*F*(3, 43) = 3.56, *p* = 0.016, η^2^*_p_* = 0.076]. Post-hoc comparisons revealed that in all conditions, SZ showed decreased abilities in choosing the correct ending to the story (Figure 4A): Physical1 [*t*(45.15) = 3.74, *p* < 0.001], ToM [*t*(37.04) = 5.01, *p* < 0.001], Physical2 [*t*(35.42) = 4.62, *p* < 0.001] and Empathy [*t*(48) = 7.52, *p* < 0.001]. In addition, we calculated the effect sizes of these comparisons, and found a larger Cohen’s d for ToM (1.45) compared to Physical1 (1.07) and for Empathy (2.07) compared to Physical2 (1.35). Further support for the greater difficulty of SZ in social cognition relative to physical control came from post-hoc comparisons between the conditions, with significant differences among this group for the pairs ToM-Physical1 [*t*(21) = 3.25, *p* = 0.004] and Empathy-Physical2 [*t*(21) = 3.32, *p* = 0.003]. For HC the ToM-Physical1 comparison was also significant [*t*(27) = 3.13, *p* = 0.004], but with a smaller mean difference (μ = 0.133 in HC vs. μ = 0.063 in SZ).

#### Response time

We analyzed the response times of correct responses using a 2 (group: SZ, HC) × 4 (condition: ToM, Physical1, Empathy, Physical2) mixed ANOVA with age, gender, and years of education as covariates (Supplementary Table S6). The results are outlined in Figure 4. Neither the main effects nor the interactions reached statistical significance (*p* ≥ 0.073).

### Emotional voices

#### Accuracy

**Figure 5:**
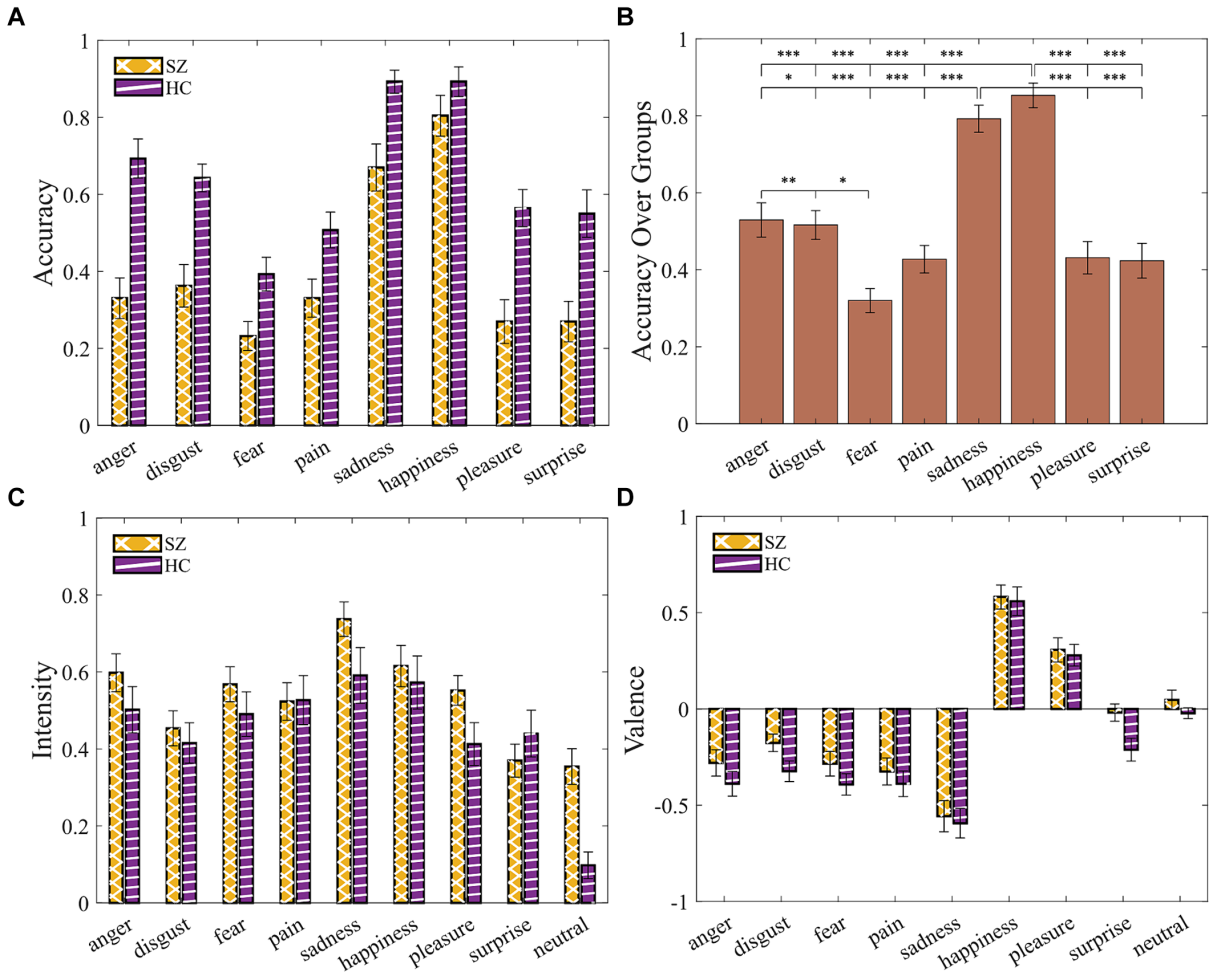
SZ exhibited impairments in identifying vocal emotions. **(A)** Accuracy by group and emotion. **(B)** Accuracy over groups demonstrates all possible pairwise comparisons, given the main effect of emotions. **(C)** Intensity ratings. **(D)** Valence ratings. Error bars indicate the standard error of the mean (SEM). Asterisks indicate level of statistical significance: *p < 0.05, **p < 0.005, and ***p < 0.001.

A 2 (group: SZ, HC) × 8 (emotion: Anger, Disgust, Fear, Pain, Sadness, Happiness, Pleasure, Surprise) mixed ANOVA with age, gender, and years of education as covariates (Supplementary Table S7) resulted in a significant main effect of group [*F*(1, 44) = 19.77, *p* < 0.001, η^2^*_p_* = 0.31], suggesting impairments in emotion recognition in SZ group (Figure 5A). The results suggested that some emotions were easier to identify given a significant main effect of emotion [*F*(7, 308) = 2.24, *p* = 0.031, η^2^*_p_* = 0.048]. Post-hoc pairwise Bonferroni-corrected comparison (Figure 5B; Supplementary Table S8) revealed that “happiness” and “sadness” were significantly more identifiable compared with all other displayed emotions. “Disgust” was also statistically different from “anger” and “fear.”

#### Intensity and valence

A 2 (group: SZ, HC) × 9 (emotion: Anger, Disgust, Fear, Pain, Sadness, Happiness, Pleasure, Surprise, Neutral) mixed ANOVA for intensity and for valence with age, gender, and years of education as covariates yielded no significant results (Supplementary Tables S9, S10; Figures 5C,D).

### Go-NoGo

**Figure 6:**
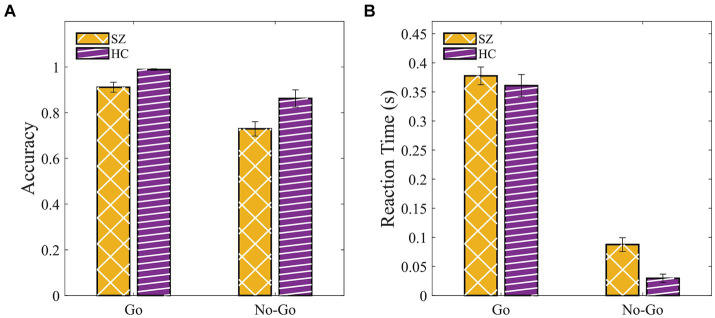
SZ were slower and tend to be less accurate compared to HC in the Go-NoGo paradigm. **(A)** Accuracy. **(B)** Response time in Go (hit) and NoGo (commission error). Error bars indicate the standard error of the mean (SEM).

We conducted a 2 (groups: SZ, HC) × 2 (condition: Go, NoGo) mixed ANOVA with age, gender, and years of education as covariates for both behavioral measures (Supplementary Tables S11, S12). We found a significant main effect of the group for RT [*F*(1, 43) = 8.38, *p* = 0.006, η^2^*_p_* = 0.163] indicating a slower response of SZ compared with HC. A similar trend was found for accuracy [*F*(1, 43) = 3.3, *p* = 0.076, η^2^*_p_* = 0.071]. Together, the Go-NoGo results suggested that SZ committed fewer hits (pressing the button in response to the “Go” signal) and more commission errors (pressing the button in response to the “NoGo” signal) compared to HC (Figures 6A,B).

## Discussion

Modified versions of three behavioral tasks that measure cognitive deficits and social cognition impairments in individuals with schizophrenia (SZ), were validated in this study. The results showed decreased performance of SZ subjects in many of the evaluated variables. They demonstrated the ability of the modified tasks to distinguish between the social cognition of SZ and HC. SZ showed a decreased ability to choose the correct endings to comic strip stories, particularly in the social cognition conditions that included ToM and empathy. Comparison between HC and SZ revealed that individuals with schizophrenia struggled to identify emotions from their vocal representation. In the measure of general (non-social) cognition, we found a slower RT of the SZ but the difference in accuracy was not significant.

The results of our study align with existing literature, highlighting the significant impairments in social cognition among individuals with schizophrenia, particularly empathy, ToM, and emotion recognition. Research has shown that both affective (Bonfils et al., 2016) and cognitive empathy (Sparks et al., 2010; Smith et al., 2012) are impaired in individuals with schizophrenia. Most studies used self-rating questionnaires to measure empathy, but these tools may not fully capture important aspects of the empathy process. Empathy is influenced not only by the perceiver but also by factors such as the target’s role and characteristics (e.g., gender and expressivity) and the situation’s valence (Zaki and Ochsner, 2009). To address this, van Donkersgoed et al. (2019) used the Empathy Accuracy Task (EAT), where emotionally charged stories are presented through video clips, alongside several questionnaires. They found that individuals with schizophrenia benefited less from others’ emotional expressivity compared to healthy controls, contributing to impaired empathic accuracy. They also noted a lack of correlation between task-based and questionnaire-based empathy measures, suggesting that self-report empathy differs from actual empathy performance (van Donkersgoed et al., 2019). These findings emphasize the importance of using objective measures, such as the stories we used in our study, to estimate social cognition competence accurately.

Studies examining ToM in schizophrenia consistently demonstrate prevalent deficits, as measured by tasks such as false belief (including verbal, picture sequencing, and story completion), deception, visual mental state jokes, irony, metaphor, hinting, and intention stories (verbal and visual) (Yeh et al., 2021). These deficits are linked to a reduced capacity to effectively engage in communication (Sperber and Wilson, 2002) and failure to monitor one’s own and others’ mental state and behavior (Frith, 2014b), in subjects with schizophrenia. However, some aspects of ToM in schizophrenia remain debated, such as whether these deficits are a state or a trait of the disease, and the association between ToM deficits and specific symptoms. Harrington et al. (2005) reviewed 30 studies exploring ToM in people with schizophrenia and found their ToM deficits are independent of general intelligence, memory, and executive functioning. Thirteen of the 30 studies examined involved other psychiatric groups as control, and in 12 of them schizophrenia groups scored significantly lower than psychiatric controls (Harrington et al., 2005). A more recent review suggests that ToM performance is influenced by neurocognition, cognitive bias, and schizophrenia symptoms (Thibaudeau et al., 2020). To better understand the association between ToM and the symptom dimensions of schizophrenia, Thibaudeau et al. (2023) conducted another review. They surveyed 130 studies and concluded that ToM is most strongly related to the Cognitive/Disorganization symptom dimension, followed by the Negative dimension. Evidence accumulated over the years has shown a connection between ToM and negative symptoms. For instance, patients with negative symptoms such as avolition or social withdrawal perform worse on ToM tasks, while those with thought insertion, delusions of alien control, or in remission perform relatively normally (Corcoran et al., 1995). Other studies indicate that individuals with severe negative symptoms tend to under-interpret the mental states of others (Peyroux et al., 2019). Additionally, ToM abilities may decline with long-duration disorders (Drury et al., 1998; Sarfati et al., 2000), as do negative symptoms (Millan et al., 2014).

Impaired social perception and cognition are crucial for understanding social behavioral problems in schizophrenia and are more predictive of diagnosis and prognosis than nonsocial cognition (Brüne, 2005). Research on social perception, or emotion perception and recognition, focuses on visual and auditory modalities (Edwards et al., 2002). In the visual modality, the prevalent approach is to use facial expressions to measure emotion recognition (Ryumina and Karpov, 2020). Such studies have consistently shown that facial emotion recognition is a common cognitive impairment in schizophrenia (Gao et al., 2021). For auditory emotion perception, emotional prosody (the intonation pattern of speech, including word stress, pauses, and lengthening of final rounds in words, Wingfield et al., 1989) is commonly used to assess recognition abilities. A recent meta-analysis of 18 studies on auditory emotional recognition found moderate deficits in schizophrenia (Gong et al., 2021), though only one study used non-verbal emotional cues (Rossell and Boundy, 2005). The reliance on prosody may explain the relatively small effect, as performance can be influenced by linguistic function, semantic content, and lack of standardized tasks (Edwards et al., 2002). Non-verbal emotional cues can potentially overcome these difficulties. In our study, we used the non-verbal MAV, incorporating all emotions and analyzing them individually. This approach is crucial given the differential relationship between specific emotions and schizophrenia symptoms, with deficits in recognizing sadness, anger, and disgust being positively associated with negative symptoms (Gong et al., 2021).

Several limitations of our study should be noted. First, the sample size was relatively small (N_SZ_ = 25; N_HC_ = 29) which may impact the interpretability of the results. For instance, the lack of statistical significance in the general cognition measure (using the Go-NoGo task) could be due to the characteristics of the task and the sample. A larger sample size, with more participants and/or trials, might yield significant differences for measures that only approached statistical significance in our study. Second, a psychiatric evaluation was conducted by the resident who collected the SZ subjects’ data to ensure they could cooperate and complete the behavioral tasks properly. However, data on their pharmacological treatment was not documented. Although the effect of antipsychotics on social cognition remains inconclusive—likely due to inconsistencies in study designs and medication dosages (Kucharska-Pietura and Mortimer, 2013)—there are cautious claims that antipsychotics may improve social cognition performance (Riccardi et al., 2021). Still, pharmacological treatment should be considered when collecting measures of social cognition in psychiatric patients. Third, there was a significant difference in the years of education between the two groups, with a larger mean number for the HC group compared with the SZ group. To estimate group differences beyond the contribution of education, we performed statistical analyses with education as a covariate. In some measures (e.g., emotional voices valence) the interaction with education was significant while in others (e.g., comics response time), it approached significance. Furthermore, many participants in the HC group were university students familiar with computerized tasks and academic research. This familiarity could partially explain the differences in social cognition measures between SZ and HC. However, considering the non-significant differences in the non-social cognition task, the disadvantages faced by the SZ group in terms of education and prior experience, and the significant results in the social cognition measures, the strength of these tasks in assessing social cognition abilities is further supported. In future research, it would be beneficial to recruit a larger, well-matched sample and to document pharmacological treatment to avoid confounding factors that could influence the interpretation of the results.

Validated social cognition measures that distinguish between individuals with schizophrenia and healthy controls can provide important tools to explore the neural correlates of social cognition impairments and negative symptoms of schizophrenia. The basal ganglia are leading candidates for such research, as they are involved in schizophrenia pathophysiology (Busatto and Kerwin, 1997) and play a role in social cognition processes. Impairments in ToM and social perception are found in many basal ganglia movement disorders (Péron et al., 2010; Pierce and Péron, 2020; Rizzo et al., 2023), and basal ganglia structures are involved in emotional prosody decoding, with structural and functional connectivity to the brain network involved in vocal emotions (Brück et al., 2011; Péron et al., 2015). Evidence for the role of this deep brain structure in social cognition in humans is made possible by innovative invasive therapeutic techniques, specifically DBS used to treat individuals in the advanced stages of Parkinson’s disease. Interestingly, there are some resemblances between Parkinson’s disease and schizophrenia. First, the symptoms of both diseases can be classified into positive and negative symptoms. The positive symptoms of Parkinson’s disease include rigidity, tremor, dystonia, akathisia, and even levodopa-induced dyskinesia and hallucination. Akinesia would be considered a negative symptom, resembling schizophrenic apathy, anhedonia, affective flattening, and mainly avolition—the lack of internal drive or motivation to act and behave—which are commonly reported in Parkinson’s patients as well. Second, “overtreating” Parkinson’s disease with dopamine replacement therapy can cause psychotic symptoms, and antipsychotic drugs may cause parkinsonian (extrapyramidal) side effects. Given these shared features, it seems reasonable to propose DBS as a novel and potentially beneficial treatment approach for schizophrenia.

We believe that using the validated battery presented here, together with future electrophysiological recording, could help improve our understanding of the basal ganglia role in social cognition, provide evidence to help settle the long-standing TT-ST debate, and lead to the development of a novel therapeutic approach (e.g., DBS) for the negative symptoms and social dysfunctions of schizophrenia.

## Data availability statement

The raw data supporting the conclusions of this article will be made available by the authors, without undue reservation.

## Ethics statement

The studies involving humans were approved by the Institutional review board of ethical conduct of the Hebrew University of Jerusalem and the Jerusalem Mental Health Center (IRB number 7-19). The studies were conducted in accordance with the local legislation and institutional requirements. The participants provided their written informed consent to participate in this study.

## Author contributions

NR: Writing – review & editing, Writing – original draft, Visualization, Software, Methodology, Investigation, Formal analysis, Data curation, Conceptualization. RG: Writing – review & editing, Data curation. OL: Writing – review & editing, Data curation. HB: Writing – review & editing, Supervision, Funding acquisition, Conceptualization. KA: Writing – review & editing. YF: Writing – review & editing. RE: Writing – review & editing, Supervision, Funding acquisition, Conceptualization.

## References

[ref1] Achim A. M. Ouellet R. Roy M. A. Jackson P. L. (2011). Assessment of empathy in first-episode psychosis and meta-analytic comparison with previous studies in schizophrenia. Psychiatry Res. 190, 3–8. doi: 10.1016/j.psychres.2010.10.030, PMID: 21131057

[ref9001] Adolphs R. (2001). The neurobiology of social cognition.” Curr Opin Neurobiol 11, 231–239. doi: 10.1016/S0959-4388(00)00202-6 11301245

[ref2] Alcalá-López D. Vogeley K. Binkofski F. Bzdok D. (2019). Building blocks of social cognition: Mirror, mentalize, share? Cortex 118, 4–18. doi: 10.1016/j.cortex.2018.05.006, PMID: 29903609

[ref3] Alonso P. Cuadras D. Gabriëls L. Denys D. Goodman W. Greenberg B. D.. (2015). Deep brain stimulation for obsessive-compulsive disorder: a meta-analysis of treatment outcome and predictors of response. PLoS One 10:e0133591. doi: 10.1371/journal.pone.0133591, PMID: 26208305 PMC4514753

[ref9002] American Psychiatric Association. (2013). Diagnostic and Statistical Manual of Mental Disorders. American Psychiatric Association.

[ref4] Apperly I. A. (2008). Beyond simulation-theory and theory-theory: why social cognitive neuroscience should use its own concepts to study “theory of mind.”. Cognition 107, 266–283. doi: 10.1016/j.cognition.2007.07.019 17868666

[ref5] Baldermann J. C. Schüller T. Huys D. Becker I. Timmermann L. Jessen F.. (2016). Deep brain stimulation for Tourette-syndrome: a systematic review and meta-analysis. Brain Stimul. 9, 296–304. doi: 10.1016/j.brs.2015.11.005, PMID: 26827109

[ref6] Baron-Cohen S. Wheelwright S. Hill J. Raste Y. Plumb I. (2001). The “Reading the mind in the eyes” test revised version: a study with Normal adults, and adults with Asperger syndrome or high-functioning autism. J. Child Psychol. Psychiatry 42, 241–251. doi: 10.1111/1469-7610.00715 11280420

[ref7] Beck A. T. Steer R. A. Carbin M. G. (1988). Psychometric properties of the Beck depression inventory: twenty-five years of evaluation. Clin. Psychol. Rev. 8, 77–100. doi: 10.1016/0272-7358(88)90050-5

[ref8] Beck A. T. Ward C. H. Mendelson M. Mock J. Erbaugh J. (1961). An inventory for measuring depression. Arch. Gen. Psychiatry 4, 561–571. doi: 10.1001/archpsyc.1961.01710120031004 13688369

[ref9003] Bellack A. S. Morrison R. L. Wixted J. T. Mueser K. T. (1990). An analysis of social competence in schizophrenia. BJPsych 156, 809–818. doi: 10.1192/bjp.156.6.809 2207511

[ref9] Belin P. Fillion-Bilodeau S. Gosselin F. (2008). The Montreal affective voices: a validated set of nonverbal affect bursts for research on auditory affective processing. Behav. Res. Methods 40, 531–539. doi: 10.3758/BRM.40.2.531 18522064

[ref10] Bernard B. A. Goldman J. G. (2010). “MMSE - mini-mental state examination” in Encyclopedia of movement disorders (Elsevier), 187–189.

[ref9004] Bodden M. E. Dodel R. Kalbe E. (2010). Theory of mind in Parkinson’s disease and related basal ganglia disorders: A systematic review. Movement Disorders. Sci. 25, 13–27. doi: 10.1002/mds.22818 19908307

[ref11] Bonfils K. A. Lysaker P. H. Minor K. S. Salyers M. P. (2016). Affective empathy in schizophrenia: a meta-analysis. Schizophr. Res. 175, 109–117. doi: 10.1016/j.schres.2016.03.037 27094715

[ref12] Brayne C. (1998). The mini-mental state examination, will we be using it in 2001? Int. J. Geriatr. Psychiatry 13, 285–290. doi: 10.1002/(sici)1099-1166(199805)13:5<285:aid-gps753>3.0.co;2-v 9658260

[ref13] Brück C. Wildgruber D. Kreifelts B. Krüger R. Wächter T. (2011). Effects of subthalamic nucleus stimulation on emotional prosody comprehension in Parkinson’s disease. PLoS One 6:e19140. doi: 10.1371/journal.pone.0019140, PMID: 21552518 PMC3084266

[ref14] Brüne M. (2005). Emotion recognition, “theory of mind,” and social behavior in schizophrenia. Psychiatry Res. 133, 135–147. doi: 10.1016/j.psychres.2004.10.007 15740990

[ref15] Brüne M. Lissek S. Fuchs N. Witthaus H. Peters S. Nicolas V.. (2008). An fMRI study of theory of mind in schizophrenic patients with “passivity” symptoms. Neuropsychologia 46, 1992–2001. doi: 10.1016/j.neuropsychologia.2008.01.023, PMID: 18329671

[ref16] Buchanan T. W. Lutz K. Mirzazade S. Specht K. Shah N. J. Zilles K.. (2000). Recognition of emotional prosody and verbal components of spoken language: an fMRI study. Brain Res. Cogn. Brain Res. 9, 227–238. doi: 10.1016/s0926-6410(99)00060-9 10808134

[ref17] Busatto G. F. Kerwin R. W. (1997). Schizophrenia, psychosis, and the basal ganglia. Psychiatr. Clin. N. Am. 20, 897–910. doi: 10.1016/S0193-953X(05)70351-8 9443356

[ref18] Carlson S. M. Koenig M. A. Harms M. B. (2013). Theory of mind. Wiley Interdiscip. Rev. Cogn. Sci. 4, 391–402. doi: 10.1002/wcs.1232 26304226

[ref9006] Christidi F. Migliaccio R. Santamaría-García H. Santangelo G. Trojsi F. (2018). Social cognition dysfunctions in neurodegenerative diseases: Neuroanatomical correlates and clinical implications. Behavioural Neurology. doi: 10.1155/2018/1849794 PMC5944290 29854017

[ref19] Corcoran R. Mercer G. Frith C. D. (1995). Schizophrenia, symptomatology and social inference: investigating “theory of mind” in people with schizophrenia. Schizophr. Res. 17, 5–13. doi: 10.1016/0920-9964(95)00024-G 8541250

[ref20] Couture S. M. Penn D. L. Roberts D. L. (2006). The functional significance of social cognition in schizophrenia: a review. Schizophr. Bull. 32, S44–S63. doi: 10.1093/schbul/sbl029, PMID: 16916889 PMC2632537

[ref21] Criaud M. Boulinguez P. (2013). Have we been asking the right questions when assessing response inhibition in go/no-go tasks with fMRI? A meta-analysis and critical review. Neurosci. Biobehav. Rev. 37, 11–23. doi: 10.1016/j.neubiorev.2012.11.003 23164813

[ref22] Cuff B. M. P. Brown S. J. Taylor L. Howat D. J. (2016). Empathy: a review of the concept. Emot. Rev. 8, 144–153. doi: 10.1177/1754073914558466

[ref23] Diamond A. (2013). Executive functions. Annu. Rev. Psychol. 64, 135–168. doi: 10.1146/annurev-psych-113011-143750, PMID: 23020641 PMC4084861

[ref24] Dodell-Feder D. Tully L. M. Hooker C. I. (2015). Social impairment in schizophrenia: new approaches for treating a persistent problem. Curr. Opin. Psychiatry 28, 236–242. doi: 10.1097/YCO.0000000000000154 25768085 PMC4478051

[ref25] Drury V. M. Robinson E. J. Birchwood M. (1998). ‘Theory of mind’ skills during an acute episode of psychosis and following recovery. Psychol. Med. 28, 1101–1112. doi: 10.1017/S0033291798006850, PMID: 9794017

[ref26] Edwards J. Jackson H. J. Pattison P. E. (2002). Emotion recognition via facial expression and affective prosody in schizophrenia. Clin. Psychol. Rev. 22, 789–832. doi: 10.1016/S0272-7358(02)00130-7 12214327

[ref27] Eitan R. Shamir R. R. Linetsky E. Rosenbluh O. Moshel S. Ben-Hur T.. (2013). Asymmetric right/left encoding of emotions in the human subthalamic nucleus. Front. Syst. Neurosci. 7:69. doi: 10.3389/fnsys.2013.00069, PMID: 24194703 PMC3810611

[ref28] Ferreira B. L. C. Fabrício D. M. Chagas M. H. N. (2021). Are facial emotion recognition tasks adequate for assessing social cognition in older people? A review of the literature. Arch. Gerontol. Geriatr. 92:104277. doi: 10.1016/j.archger.2020.104277 33091714

[ref29] Ferretti V. Papaleo F. (2019). Understanding others: emotion recognition in humans and other animals. Genes Brain Behav. 18:e12544. doi: 10.1111/gbb.12544 30549185

[ref30] Fett A. K. J. Viechtbauer W. Dominguez G. M. Penn D. L. van Os J. Krabbendam L. (2011). The relationship between neurocognition and social cognition with functional outcomes in schizophrenia: a meta-analysis. Neurosci. Biobehav. Rev. 35, 573–588. doi: 10.1016/j.neubiorev.2010.07.001, PMID: 20620163

[ref31] Folstein M. F. Folstein S. E. Mchugh P. R. (1975). “Mini-mental state”: a practical method for grading the cognitive state of patients for the clinician. J. Psychiatr. Res. 12, 189–198. doi: 10.1016/0022-3956(75)90026-6 1202204

[ref32] Frith C. D. (2014). The cognitive neuropsychology of schizophrenia. 1st Edn. London: Psychology Press.

[ref33] Frith C. Frith U. (2005). Theory of mind. Curr. Biol. 15, R644–R645. doi: 10.1016/j.cub.2005.08.041 16139190

[ref34] Galderisi S. Kaiser S. Bitter I. Nordentoft M. Mucci A. Sabé M.. (2021). EPA guidance on treatment of negative symptoms in schizophrenia. Eur. Psychiatry 64:e21. doi: 10.1192/j.eurpsy.2021.13 33726883 PMC8057437

[ref35] Gao Z. Zhao W. Liu S. Liu Z. Yang C. Xu Y. (2021). Facial emotion recognition in schizophrenia. Front. Psych. 12:633717. doi: 10.3389/fpsyt.2021.633717, PMID: 34017272 PMC8129182

[ref36] Gong B. Li Q. Zhao Y. Wu C. (2021). Auditory emotion recognition deficits in schizophrenia: a systematic review and meta-analysis. Asian J. Psychiatr. 65:102820. doi: 10.1016/j.ajp.2021.102820, PMID: 34482183

[ref37] Graat I. Figee M. Denys D. (2017). The application of deep brain stimulation in the treatment of psychiatric disorders. Int. Rev. Psychiatry 29, 178–190. doi: 10.1080/09540261.2017.1282439 28523977

[ref9005] Grèzes J. Frith C. D. Passingham R. E. (2004). Inferring false beliefs from the actions of oneself and others: an fMRI study. Neuroimage. 21, 744–750. doi: 10.1016/j.neuroimage.2003.10.014 14980577

[ref38] Haddad P. M. Correll C. U. (2018). The acute efficacy of antipsychotics in schizophrenia: a review of recent meta-analyses. Ther. Adv. Psychopharmacol. 8, 303–318. doi: 10.1177/2045125318781475 30344997 PMC6180374

[ref39] Harrington L. Siegert R. J. McClure J. (2005). Theory of mind in schizophrenia: a critical review. Cogn. Neuropsychiatry 10, 249–286. doi: 10.1080/13546800444000056 16571462

[ref40] Hoekert M. Kahn R. S. Pijnenborg M. Aleman A. (2007). Impaired recognition and expression of emotional prosody in schizophrenia: review and meta-analysis. Schizophr. Res. 96, 135–145. doi: 10.1016/j.schres.2007.07.023 17766089

[ref41] Horan W. P. Jimenez A. M. Lee J. Wynn J. K. Eisenberger N. I. Green M. F. (2016). Pain empathy in schizophrenia: an fMRI study. Soc. Cogn. Affect. Neurosci. 11, 783–792. doi: 10.1093/SCAN/NSW002 26746181 PMC4847698

[ref42] Kahn R. S. Sommer I. E. Murray R. M. Meyer-Lindenberg A. Weinberger D. R. Cannon T. D.. (2015). Schizophrenia. Nat. Rev. Dis. Primers 1:15067. doi: 10.1038/nrdp.2015.67 27189524

[ref43] Kalin M. Kaplan S. Gould F. Pinkham A. E. Penn D. L. Harvey P. D. (2015). Social cognition, social competence, negative symptoms and social outcomes: inter-relationships in people with schizophrenia. J. Psychiatr. Res. 68, 254–260. doi: 10.1016/j.jpsychires.2015.07.008 26228427 PMC4524806

[ref44] Klosterkötter J. (2008). Indicated prevention of schizophrenia. Dtsch. Arztebl. Int. 105, 532–539. doi: 10.3238/arztebl.2008.0532 19626210 PMC2696964

[ref45] Kotov R. Fochtmann L. Li K. Tanenberg-Karant M. Constantino E. A. Rubinstein J.. (2017). Declining clinical course of psychotic disorders over the two decades following first hospitalization: evidence from the Suffolk county mental health project. Am. J. Psychiatry 174, 1064–1074. doi: 10.1176/appi.ajp.2017.16101191, PMID: 28774193 PMC5767161

[ref46] Kraus M. S. Walker T. M. Jarskog L. F. Millet R. A. Keefe R. S. E. (2019). Basic auditory processing deficits and their association with auditory emotion recognition in schizophrenia. Schizophr. Res. 204, 155–161. doi: 10.1016/j.schres.2018.08.031 30268821

[ref47] Kucharska-Pietura K. Mortimer A. (2013). Can antipsychotics improve social cognition in patients with schizophrenia? CNS Drugs 27, 335–343. doi: 10.1007/s40263-013-0047-0 23533009 PMC3657085

[ref9007] Lamm C. Decety J. Singer T. (2011). Meta-analytic evidence for common and distinct neural networks associated with directly experienced pain and empathy for pain. Neuroimage. 54, 2492–2502. doi: 10.1016/j.neuroimage.2010.10.014, PMID: 20946964

[ref48] Langdon R. Coltheart M. Ward P. B. Catts S. V. (2001). Mentalising, executive planning and disengagement in schizophrenia. Cogn. Neuropsychiatry 6, 81–108. doi: 10.1080/13546800042000061

[ref49] Lee S. J. Kang D. H. Kim C. W. Gu B. M. Park J. Y. Choi C. H.. (2010). Multi-level comparison of empathy in schizophrenia: an fMRI study of a cartoon task. Psychiatry Res. Neuroimaging 181, 121–129. doi: 10.1016/j.pscychresns.2009.08.003 20080395

[ref50] Lin Y. Ding H. Zhang Y. (2018). Emotional prosody processing in schizophrenic patients: a selective review and meta-analysis. J. Clin. Med. 7:363. doi: 10.3390/JCM7100363, PMID: 30336573 PMC6210777

[ref51] Madeira N. Caldeira S. Bajouco M. Pereira A. T. Martins M. J. Macedo A. (2016). Social cognition, negative symptoms and psychosocial functioning in schizophrenia. Int. J. Clin. Neurosci. Ment. Health 2016:1. doi: 10.21035/ijcnmh.2016.3.1

[ref52] Malone D. A. (2010). Use of deep brain stimulation in treatment-resistant depression. Cleve. Clin. J. Med. 77, S77–S80. doi: 10.3949/ccjm.77.s3.14 20622083

[ref53] Marmor O. Rappel P. Valsky D. Bick A. S. Arkadir D. Linetsky E.. (2020). Movement context modulates neuronal activity in motor and limbic-associative domains of the human parkinsonian subthalamic nucleus. Neurobiol. Dis. 136:104716. doi: 10.1016/j.nbd.2019.104716 31846735

[ref54] Michaels T. M. Horan W. P. Ginger E. J. Martinovich Z. Pinkham A. E. Smith M. J. (2014). Cognitive empathy contributes to poor social functioning in schizophrenia: evidence from a new self-report measure of cognitive and affective empathy. Psychiatry Res. 220, 803–810. doi: 10.1016/j.psychres.2014.08.054 25632418

[ref55] Millan M. J. Fone K. Steckler T. Horan W. P. (2014). Negative symptoms of schizophrenia: clinical characteristics, pathophysiological substrates, experimental models and prospects for improved treatment. Eur. Neuropsychopharmacol. 24, 645–692. doi: 10.1016/j.euroneuro.2014.03.008 24820238

[ref56] Pell M. D. (2006). Cerebral mechanisms for understanding emotional prosody in speech. Brain Lang. 96, 221–234. doi: 10.1016/j.bandl.2005.04.007, PMID: 15913754

[ref57] Péron J. Frühholz S. Ceravolo L. Grandjean D. (2015). Structural and functional connectivity of the subthalamic nucleus during vocal emotion decoding. Soc. Cogn. Affect. Neurosci. 11, 349–356. doi: 10.1093/scan/nsv118 26400857 PMC4733346

[ref58] Péron J. le Jeune F. Haegelen C. Dondaine T. Drapier D. Sauleau P.. (2010). Subthalamic nucleus stimulation affects theory of mind network: a PET study in Parkinson’s disease. PLoS One 5:e9919. doi: 10.1371/journal.pone.0009919, PMID: 20360963 PMC2847915

[ref59] Peyroux E. Prost Z. Danset-Alexandre C. Brenugat-Herne L. Carteau-Martin I. Gaudelus B.. (2019). From “under” to “over” social cognition in schizophrenia: is there distinct profiles of impairments according to negative and positive symptoms? Schizophr. Res. Cogn. 15, 21–29. doi: 10.1016/j.scog.2018.10.001 30534527 PMC6260279

[ref60] Pierce J. E. Péron J. (2020). The basal ganglia and the cerebellum in human emotion. Soc. Cogn. Affect. Neurosci. 15, 599–613. doi: 10.1093/scan/nsaa076, PMID: 32507876 PMC7328022

[ref61] Riccardi C. Montemagni C. Del Favero E. Bellino S. Brasso C. Rocca P. (2021). Pharmacological treatment for social cognition: current evidence. Int. J. Mol. Sci. 22:7457. doi: 10.3390/ijms22147457, PMID: 34299076 PMC8307511

[ref62] Rizzo G. Martino D. Avanzino L. Avenanti A. Vicario C. M. (2023). Social cognition in hyperkinetic movement disorders: a systematic review. Soc. Neurosci. 18, 331–354. doi: 10.1080/17470919.2023.2248687 37580305

[ref63] Robinson D. G. Woerner M. G. McMeniman M. Alan Mendelowitz M. Bilder R. M. (2004). Symptomatic and functional recovery from a first episode of schizophrenia or schizoaffective disorder. Am. J. Psychiatry 161, 473–479. doi: 10.1176/appi.ajp.161.3.473 14992973

[ref64] Rossell S. L. Boundy C. L. (2005). Are auditory–verbal hallucinations associated with auditory affective processing deficits? Schizophr. Res. 78, 95–106. doi: 10.1016/j.schres.2005.06.002 16005614

[ref65] Ryumina E. V. Karpov A. A. (2020). Analytical review of methods for emotion recognition by human face expressions. Sci. Tech. J. Inform. Technol. Mech. Opt. 20, 163–176. doi: 10.17586/2226-1494-2020-20-2-163-176

[ref66] Sarfati Y. Passerieux C. Hardy-Baylé M.-C. (2000). Can verbalization remedy the theory of mind deficit in schizophrenia? Psychopathology 33, 246–251. doi: 10.1159/000029153 10965281

[ref67] Savla G. N. Vella L. Armstrong C. C. Penn D. L. Twamley E. W. (2013). Deficits in domains of social cognition in schizophrenia: a meta-analysis of the empirical evidence. Schizophr. Bull. 39, 979–992. doi: 10.1093/schbul/sbs080 22949733 PMC3756768

[ref68] Scherer K. R. Ladd D. R. Silverman K. E. A. (1984). Vocal cues to speaker affect: testing two models. J. Acoust. Soc. Am. 76, 1346–1356. doi: 10.1121/1.391450

[ref69] Shamay-Tsoory S. G. (2013). “Empathic processing: its cognitive and affective dimensions and neuroanatomical basis” in The social neuroscience of empathy. Boston: (The MIT Press), 215–232.

[ref9009] Shamay-Tsoory S. G. Aharon-Peretz J. Perry D. (2009). Two systems for empathy: A double dissociation between emotional and cognitive empathy in inferior frontal gyrus versus ventromedial prefrontal lesions. Brain 132, 617–627. doi: 10.1093/brain/awn279 18971202

[ref70] Shamay-Tsoory S. G. Shur S. Harari H. Levkovitz Y. (2007). Neurocognitive basis of impaired empathy in schizophrenia. Neuropsychology 21, 431–438. doi: 10.1037/0894-4105.21.4.431 17605576

[ref71] Shamay-Tsoory S. G. Tomer R. Goldsher D. Berger B. D. Aharon-Peretz J. (2004). Impairment in cognitive and affective empathy in patients with brain lesions: anatomical and cognitive correlates. J. Clin. Exp. Neuropsychol. 26, 1113–1127. doi: 10.1080/13803390490515531 15590464

[ref72] Smith M. J. Horan W. P. Karpouzian T. M. Abram S. V. Cobia D. J. Csernansky J. G. (2012). Self-reported empathy deficits are uniquely associated with poor functioning in schizophrenia. Schizophr. Res. 137, 196–202. doi: 10.1016/j.schres.2012.01.012 22321668

[ref73] Sparks A. McDonald S. Lino B. O’Donnell M. Green M. J. (2010). Social cognition, empathy and functional outcome in schizophrenia. Schizophr. Res. 122, 172–178. doi: 10.1016/j.schres.2010.06.011 20609567

[ref74] Sperber D. Wilson D. (2002). Pragmatics, modularity and mind-reading. Mind Lang. 17, 3–23. doi: 10.1111/1468-0017.00186

[ref75] Swartz M. S. Perkins D. O. Stroup T. S. Davis S. M. Capuano G. Rosenheck R. A.. (2007). Effects of antipsychotic medications on psychosocial functioning in patients with chronic schizophrenia: findings from the NIMH CATIE study. Am. J. Psychiatry 164, 428–436. doi: 10.1176/ajp.2007.164.3.428, PMID: 17329467

[ref76] Tamminga C. A. Buchanan R. W. Gold J. M. (1998). The role of negative symptoms and cognitive dysfunction in schizophrenia outcome. Int. Clin. Psychopharmacol. 13, S21–S26. doi: 10.1097/00004850-199803003-00004 9690966

[ref77] Thibaudeau É. Achim A. M. Parent C. Turcotte M. Cellard C. (2020). A meta-analysis of the associations between theory of mind and neurocognition in schizophrenia. Schizophr. Res. 216, 118–128. doi: 10.1016/j.schres.2019.12.017 31899095

[ref78] Thibaudeau E. Rae J. Raucher-Chéné D. Bougeard A. Lepage M. (2023). Disentangling the relationships between the clinical symptoms of schizophrenia Spectrum disorders and theory of mind: a meta-analysis. Schizophr. Bull. 49, 255–274. doi: 10.1093/schbul/sbac150 36244001 PMC10016420

[ref79] Trommer B. L. Hoeppner J. B. Lorber R. Armstrong K. J. (1988). The Go-No-Go paradigm in attention deficit disorder. Ann. Neurol. 24, 610–614. doi: 10.1002/ana.410240504 3202613

[ref9008] Van Der Werf M. Hanssen M. Köhler S. Verkaaik M. Verhey F. R. Van Winkel R.. (2014). Systematic review and collaborative recalculation of 133 693 incident cases of schizophrenia. Psychol Med. 44, 9–16. doi: 10.1017/S0033291712002796 23244442

[ref80] van Donkersgoed R. J. M. de Jong S. aan het Rot M. Wunderink L. Lysaker P. H. Hasson-Ohayon I.. (2019). Measuring empathy in schizophrenia: the empathic accuracy task and its correlation with other empathy measures. Schizophr. Res. 208, 153–159. doi: 10.1016/j.schres.2019.03.024, PMID: 31006615

[ref81] van Neerven T. Bos D. J. van Haren N. E. (2021). Deficiencies in theory of mind in patients with schizophrenia, bipolar disorder, and major depressive disorder: a systematic review of secondary literature. Neurosci. Biobehav. Rev. 120, 249–261. doi: 10.1016/j.neubiorev.2020.11.011 33246019

[ref82] Völlm B. A. Taylor A. N. W. Richardson P. Corcoran R. Stirling J. McKie S.. (2006). Neuronal correlates of theory of mind and empathy: a functional magnetic resonance imaging study in a nonverbal task. Neuroimage 29, 90–98. doi: 10.1016/j.neuroimage.2005.07.022 16122944

[ref83] Walter H. Ciaramidaro A. Adenzato M. Vasic N. Ardito R. B. Erk S.. (2009). Dysfunction of the social brain in schizophrenia is modulated by intention type: an fMRI study. Soc. Cogn. Affect. Neurosci. 4, 166–176. doi: 10.1093/scan/nsn047, PMID: 19287044 PMC2686225

[ref84] Wingfield A. Lahar C. J. Stine E. A. L. (1989). Age and decision strategies in running memory for speech: effects of prosody and linguistic structure. J. Gerontol. 44:106. doi: 10.1093/geronj/44.4.P106 2738311

[ref85] Yeh Y. C. Lin C. Y. Li P. C. Hung C. F. Cheng C. H. Kuo M. H.. (2021). A systematic review of the current measures of theory of mind in adults with schizophrenia. Int. J. Environ. Res. Public Health 18:7172. doi: 10.3390/ijerph18137172, PMID: 34281109 PMC8297277

[ref86] Zaki J. Ochsner K. (2009). The need for a cognitive neuroscience of naturalistic social cognition. Ann. N. Y. Acad. Sci. 1167, 16–30. doi: 10.1111/j.1749-6632.2009.04601.x 19580548 PMC2897139

