# Supplementary material for: Validation of behavioral measures of social cognition in individuals diagnosed with schizophrenia
Source: Front Psychol. 2024 Sep 9;15:1443145. doi: 10.3389/fpsyg.2024.1443145 (PMC11420990; doi:10.3389/fpsyg.2024.1443145)
Supplement: Supplementary file 1 [file Data_Sheet_1.docx]

SUPPLEMENTARY MATERIAL

**Table S1. Sample Characteristics Summary** Independent Samples 2-tailed t-test

| **Effect** | ***df*** | **t** | ***P*** |
| --- | --- | --- | --- |
| Gender | (52) | 2.9 | **.005** |
| Age | (51.1) | -1.48 | .144 |
| Education | (51) | 5.44 | **<.001** |

**Table S2. Number of Participants in the Different Behavioral Tasks**

| **Task** | **HC** | **SZ** |
| --- | --- | --- |
| Comics strip | 27 | 22 |
| Emotional Voices | 27 | 23 |
| Go No-Go | 26 | 23 |

**Table S3. BDI Behavioral Accuracy Correlations**

| **Variable** | ***n*** | ***M*** | ***SD*** | **R** | **p** |
| --- | --- | --- | --- | --- | --- |
| Comic Strip |  |  |  |  |  |
| ToM Accuracy | 50 | .77 | .19 | -.006 | .977 |
| Physical1 Accuracy | 50 | .86 | .17 | .082 | .704 |
| Empathy Accuracy | 50 | .74 | .19 | -.247 | .244 |
| Physical2 Accuracy | 50 | .79 | .16 | -.318 | .13 |
| Emotional Voices |  |  |  |  |  |
| Anger Accuracy | 51 | .53 | .32 | -.17 | .449 |
| Disgust Accuracy | 51 | .52 | .26 | -.426 | .048 |
| Fear Accuracy | 51 | .32 | .22 | .041 | .855 |
| Pain Accuracy | 51 | .43 | .26 | -.546 | .009 |
| Sadness Accuracy | 51 | .79 | .25 | -.039 | .864 |
| Happiness Accuracy | 51 | .85 | .23 | -.171 | .447 |
| Pleasure Accuracy | 51 | .43 | .3 | -.428 | .047 |
| Surprise Accuracy | 51 | .42 | .32 | -.459 | .032 |
| Go No-Go |  |  |  |  |  |
| Go Accuracy | 50 | .95 | .08 | -.199 | .35 |
| No-Go Accuracy | 50 | .8 | .19 | -.139 | .516 |

Bonferroni corrections were applied due to multiple comparisons. As a result, the p values required to reach statistical significance were p<0.0125 in the Comic Strip, p<0.00625 in Emotional Voices, and p<0.025 in the Go-NoGo.

**Table S4. MMSE Behavioral Accuracy Correlations**

| **Variable** | ***n*** | ***M*** | ***SD*** | **R** | **p** |
| --- | --- | --- | --- | --- | --- |
| Comic Strip |  |  |  |  |  |
| ToM Accuracy | 50 | .77 | .19 | -.033 | .875 |
| Physical1 Accuracy | 50 | .86 | .17 | .007 | .974 |
| Empathy Accuracy | 50 | .74 | .19 | -.206 | .323 |
| Physical2 Accuracy | 50 | .79 | .16 | -.122 | .561 |
| Emotional Voices |  |  |  |  |  |
| Anger Accuracy | 51 | .53 | .32 | -.028 | .895 |
| Disgust Accuracy | 51 | .52 | .26 | -.186 | .372 |
| Fear Accuracy | 51 | .32 | .22 | -.474 | .017 |
| Pain Accuracy | 51 | .43 | .26 | -.485 | .014 |
| Sadness Accuracy | 51 | .79 | .25 | -.145 | .489 |
| Happiness Accuracy | 51 | .85 | .23 | -.237 | .254 |
| Pleasure Accuracy | 51 | .43 | .3 | .063 | .764 |
| Surprise Accuracy | 51 | .42 | .32 | .208 | .317 |
| Go No-Go |  |  |  |  |  |
| Go Accuracy | 50 | .95 | .08 | .009 | .964 |
| No-Go Accuracy | 50 | .8 | .19 | .18 | .389 |

Bonferroni corrections were applied due to multiple comparisons. As a result, the p values required to reach statistical significance were p<0.0125 in the Comic Strip, p<0.00625 in Emotional Voices, and p<0.025 in the Go-NoGo.

**Table S5. Comics Strip Accuracy.** *Summary of Mixed Analysis of Variance (ANOVA) for 2 (groups: SZ and HC) × 4 (condition: Physical1, ToM, Physical2, and Empathy) with age, gender, and years of education as covariates.*

| **Effect** | ***df*** | **F** | ***P*** | **ƞ^2^*_p_*** |
| --- | --- | --- | --- | --- |
| Condition | (3,129) | .5 | .69 | .01 |
| Group | (1,43) | 12.31 | **.001** | .22 |
| Condition *×* Group | (3,129) | 3.56 | **.016** | .076 |
| Age | (1,43) | .21 | .651 | .043 |
| Education | (1,43) | .77 | .386 | .076 |
| Gender | (1,43) | .25 | .62 | .006 |

**Table S6. Comics Strip Response Time.** Summary of Mixed Analysis of Variance (ANOVA) for 2 (groups: SZ and HC) × 4 (condition: Physical1, ToM, Physical2, and Empathy) with age, gender, and years of education as covariates.

| **Effect** | ***df*** | **F** | ***P*** | **ƞ^2^*_p_*** |
| --- | --- | --- | --- | --- |
| Condition | (3,129) | .283 | .837 | .007 |
| Group | (1,43) | 3.38 | .073 | .073 |
| Condition *×* Group | (3,129) | .642 | .589 | .015 |
| Age | (1,43) | .009 | .927 | <.001 |
| Education | (1,43) | .32 | .574 | .007 |
| Gender | (1,43) | .053 | .818 | .001 |

**Table S7. Emotional Voices Accuracy.** Summary of Mixed Analysis of Variance (ANOVA) for 2 (groups: SZ and HC) × 8 (emotion: Anger, Disgust, Fear, Pain, Sadness, Neutral, Happiness, Pleasure, and Surprise) with age, gender, and years of education as covariates.

| **Effect** | ***df*** | **F** | ***P*** | **ƞ^2^*_p_*** |
| --- | --- | --- | --- | --- |
| Emotion | (7,308) | 2.24 | **.031** | .048 |
| Group | (1,44) | 19.77 | **<.001** | .31 |
| Emotion *×* Group | (7,308) | .6 | .757 | .013 |
| Age | (1,44) | <.001 | .987 | <.001 |
| Education | (1,44) | .638 | .429 | .014 |
| Gender | (1,44) | <.001 | .996 | <.001 |

**Table S8**. **Emotional Voices Accuracy Post-hoc Comparisons p-values**

|  | Disgust | Fear | Pain | Sadness | Happiness | Pleasure | Surprise |
| --- | --- | --- | --- | --- | --- | --- | --- |
| Anger | 1 | .004 | 1 | .01 | <.001 | 1 | .812 |
| Disgust |  | .042 | 1 | <.001 | <.001 | 1 | 1 |
| Fear |  |  | 1 | <.001 | <.001 | 1 | 1 |
| Pain |  |  |  | <.001 | <.001 | 1 | 1 |
| Sadness |  |  |  |  | 1 | <.001 | <.001 |
| Happiness |  |  |  |  |  | <.001 | <.001 |
| Pleasure |  |  |  |  |  |  | 1 |

**Table S9. Emotional Voices Intensity.** Summary of Mixed Analysis of Variance (ANOVA) for 2 (groups: SZ and HC) × 9 (emotion: Anger, Disgust, Fear, Pain, Sadness, Neutral, Happiness, Pleasure, and Surprise) with age, gender, and years of education as covariates.

| **Effect** | ***df*** | **F** | ***P*** | **ƞ^2^*_p_*** |
| --- | --- | --- | --- | --- |
| Emotion | (8,352) | 1.27 | .28 | .028 |
| Group | (1,44) | 1.79 | .188 | .039 |
| Emotion *×* Group | (8,352) | 1.56 | .176 | .034 |
| Age | (1,44) | .031 | .861 | .001 |
| Education | (1,44) | 1.83 | .188 | .039 |
| Gender | (1,44) | .16 | .692 | .004 |

**Table S10. Emotional Voices Valence.** Summary of Mixed Analysis of Variance (ANOVA) for 2 groups × 9 emotions with age, gender, and years of education as covariates.

| **Effect** | ***df*** | **F** | ***P*** | **ƞ^2^*_p_*** |
| --- | --- | --- | --- | --- |
| Emotion | (8,352) | 1.27 | .26 | .028 |
| Group | (1,44) | .22 | .882 | .001 |
| Emotion *×* Group | (8,352) | 1.02 | .406 | .023 |
| Age | (1,44) | 1.01 | .32 | .022 |
| Education | (1,44) | 1.64 | .207 | .036 |
| Gender | (1,44) | .23 | .633 | .005 |

**Table S11. Go-NoGo Accuracy.** Summary of Mixed Analysis of Variance (ANOVA) for 2 (groups: SZ, HC) × 2 (condition: Go, NoGo) with age, gender, and years of education as covariates.

| **Effect** | ***df*** | **F** | ***P*** | **ƞ^2^*_p_*** |
| --- | --- | --- | --- | --- |
| Condition | (1,43) | 1.74 | .194 | .039 |
| Group | (1,43) | 3.3 | .076 | .071 |
| Condition *×* Group | (1,43) | .02 | .887 | <.001 |
| Age | (1,43) | .285 | .596 | .007 |
| Education | (1,43) | 4.99 | **.031** | .104 |
| Gender | (1,43) | 2.75 | .105 | .06 |

**Table S12. Go-NoGo Response Time.** Summary of Mixed Analysis of Variance (ANOVA) for 2 (groups: SZ and HC) × 2 (condition: Go, NoGo) with age, gender, and years of education as covariates.

| **Effect** | ***df*** | **F** | ***P*** | **ƞ^2^*_p_*** |
| --- | --- | --- | --- | --- |
| Condition | (1,43) | 1.2 | .279 | .027 |
| Group | (1,43) | 8.38 | **.006** | .163 |
| Condition *×* Group | (1,43) | .04 | .851 | .001 |
| Age | (1,43) | .014 | .906 | <.001 |
| Education | (1,43) | 1.53 | .223 | .034 |
| Gender | (1,43) | 1.6 | .309 | .024 |
